# Pattern of Inflammatory Markers and Use of Antibiotics in Meconium Aspiration Syndrome: A Retrospective Cohort Study

**DOI:** 10.7759/cureus.44921

**Published:** 2023-09-08

**Authors:** Uvaraj Periasamy, Agnes Salvador, Michael Janeczko

**Affiliations:** 1 Anesthesiology and Critical Care, Boston Children's Hospital, Boston, USA; 2 Pediatrics and Neonatology, Einstein Medical Center Philadelphia, Philadelphia, USA

**Keywords:** antibiotics, c-reactive protein, inflammatory markers, neonatal intensive care unit, meconium aspiration syndrome

## Abstract

Objectives

To study the pattern of inflammatory markers in meconium aspiration syndrome (MAS) and their correlation with illness severity/antibiotic usage.

Study design

This is a retrospective analysis of neonates who were admitted with MAS and had inflammatory markers done during the first week of life.

Results

Seventy-six neonates with MAS were identified. White cell count (WCC), absolute neutrophil count (ANC), and immature to total neutrophil count (I/T) ratio peaked at 12 and CRP (43.75 mg/dl) at 48 hours of life (HOL). Neonates needing nasal cannula oxygen had lower CRP at 12 (p=0.035) and 24 HOL (p=0.046). There was no correlation between CRP at 48 HOL and score for neonatal acute physiology and perinatal extension II (SNAPPE-II; R^2 ^0.0004). High CRP at 24 HOL was associated with longer duration of antibiotics (p<0.001) despite no correlation with the blood cultures.

Conclusion

MAS was associated with inflammatory markers peaking at 12 to 48 HOL; however, antibiotics should not be determined based on them as their correlation for illness severity or blood culture is poor.

## Introduction

Meconium staining of amniotic fluid occurs in 8-15% of term deliveries [1,2] and meconium aspiration syndrome (MAS) occurs in 1.5% of neonates exposed to meconium-stained amniotic fluid [3,4]. Experimental studies have shown that meconium can induce an inflammatory response cascade in the host leading to chemical pneumonitis. Adult rabbit model lungs showed infiltration of polymorphonuclear leukocytes in alveolar septa within six hours after the instillation of meconium [5]. In a piglet model with MAS, meconium was found to be a potent activator of complement leading to an inflammatory profile that closely resembled a systemic inflammatory response syndrome [6]. Elevations in serum inflammatory markers such as C-reactive protein (CRP), white cell count (WCC), absolute neutrophil count (ANC), and immature to total neutrophil count ratio (I/T ratio) have been observed in neonates with MAS [7-10]; however, studies that clearly elucidate the pattern of these elevations are lacking.

The association between meconium aspiration and neonatal sepsis has been evaluated extensively, but the role of antibiotics in the routine management of MAS remains controversial [11,12]. Increasing evidence suggests that prolonged use of antimicrobials can lead to various undesirable outcomes for neonates, including necrotizing enterocolitis, and disseminated fungal infection [13,14]. In addition, there are potential deleterious long-term implications of altering the neonatal microbiome with injudicious antimicrobial therapy [15,16]. Our objectives are to describe the pattern of inflammatory markers during the first week of life and analyze their correlation with illness severity, respiratory support, blood culture results and duration of antibiotic use.

## Materials and methods

Study design and population

This is a human research study with minimal risks to the patients reported in the study. An Institutional Review Board approval was obtained from our institution, Einstein Medical Center Philadelphia (IRB-2020-175). This is a retrospective observational study of a cohort of neonates born from 01/01/2015 to 12/31/2018 who were admitted with MAS to a Level 3 neonatal intensive care unit (NICU) in a tertiary care hospital. Meconium aspiration syndrome was defined as respiratory distress occurring soon after birth in an infant born through a meconium-stained fluid with radiological findings compatible with MAS, including diffuse, asymmetric, patchy, or streaky infiltrates with areas of hyperinflation, consolidation, or atelectasis [9].

All neonates with the International Classification of Diseases (ICD) 9/10 codes of MAS were identified and diagnosis of MAS, as defined above, was confirmed by chart review. Inflammatory markers included were CRP, WCC, ANC, and I/T ratio. Neonates who had at least a single value of CRP, WCC, ANC, and I/T ratio during the first week of illness were included in the study. We did not include preterm neonates <37 weeks gestation since the respiratory illness in these patients may be confounded by surfactant deficiency due to prematurity. Neonates who either were transferred to other institutions or expired within 24 hours of life (HOL) were excluded from the study as we were unable to follow their inflammatory parameters.

The unit for CRP was expressed in mg/L and a value above 5 mg/L was considered as significant [17,18]. WCC and ANC were determined by an automated cell analyzer. The ANC was calculated using the formula 10 x WCC x total neutrophils (% granulocytes + % bands) and a value less than 1500 is considered significant [19]. I/T ratio was calculated as the ratio of sum of immature granulocytes (precursor cells and bands) to the sum of total granulocytes (immature and mature granulocytes) and expressed as a fraction. An I/T ratio of more than 0.2 was considered significant [19].

Data collection

We collected data from the electronic medical record (EMR) and exported it into a standardized collection form. Patient characteristics collected included sex, gestational age, type of delivery, and birth weight. Maternal factors included group B streptococcus (GBS) colonization status, prophylactic antibiotics against GBS if indicated, duration of rupture of membranes, and chorioamnionitis. We collected respiratory parameters, including intubation at birth, received surfactant therapy and maximum level of respiratory support required (nasal cannula oxygen, non-invasive and invasive mechanical ventilation). Noninvasive ventilation (NIV) included high flow nasal cannula (HFNC) more than 1L/min of oxygen [20], continuous positive airway pressure (CPAP) and non-invasive positive pressure ventilation (NIPPV). To determine the contribution of antibiotic therapy to inflammatory marker values, we collected data on blood culture results, need for lumbar puncture, cerebrospinal fluid (CSF) culture results, use of antibiotics and duration of antibiotics. Illness severity was assessed by the score for neonatal acute physiology and perinatal extension II (SNAPPE-II) [21].

Statistical analysis

Categorical variables were reported as proportions and continuous variables as median with interquartile ranges (IQR). To determine the pattern of the inflammatory markers, mean values of CRP, WCC, ANC, and I/T ratio were plotted in a graph against age in hours. Further analysis was performed with neonates who had SNAPPE-II scores available. As CRP is widely used to determine antibiotic usage in clinical practice, as a priori we decided to perform an R-squared (R2) analysis between peak CRP at 48 hours of age and SNAPPE-II score. To determine the significance of CRP, a multiple logistic regression was performed with CRP at 12, 24, 48, 72, and 96 hours of age against respiratory support, blood culture, duration of antibiotics, and SNAPPE-II score. As CRP has a higher negative predictive value, we sought to determine the association between CRP and negative blood cultures with the positive blood cultures as reference. The results were expressed as correlation coefficient with 95% confidence interval (CI) and a p value of <0.05 was considered as significant.

## Results

A total of 83 neonates with MAS were identified with the ICD 9/10 codes during the study period. Seven of them were excluded (transfer 5, death 1, and no meconium 1) with a total of 76 neonates included in the study. Neonatal and maternal characteristics are shown in Table 1. The median gestational age at birth was 40 weeks (IQR 39+2 to 41) and the median birth weight was 3350 grams (IQR 3080 to 3723). The proportion of mothers who were group B streptococcus (GBS) positive or unknown was 32% with 71% of them receiving intrapartum antibiotic prophylaxis. Chorioamnionitis was present in 18 (24%) of the mothers and prolonged rupture of membranes (PROM) was noted in 11 (14%). The mode of delivery was equally divided between spontaneous vaginal delivery and cesarean section for all the neonates.

**Table 1:** Neonatal and maternal characteristics of neonates with MAS N, number of total patients; IQR, interquartile range; NIV, noninvasive ventilation; GBS: Group B streptococcus; NICU: neonatal intensive care unit; MAS: meconium aspiration syndrome

Variables	N = 76
Sex	
Male	33 (43%)
Female	43 (57%)
Gestational age (weeks) (Median with IQR)	40 (39+2 to 41)
Birth weight (grams) (Median with IQR)	3350 (3080 to 3723)
Maternal GBS positive/unknown	24 (32%)
Maternal intrapartum prophylaxis	17/24 (71%)
Maternal chorioamnionitis	18 (24%)
Prolonged rupture of membranes	11 (14%)
Delivery	
Spontaneous vaginal delivery	38 (50%)
Cesarean section	38 (50%)
Intubation at birth	19 (25%)
Respiratory support	
Nasal cannula oxygen	4 (5%)
NIV	51 (67%)
Invasive ventilation	21 (28%)
Surfactant administration	12 (16%)
Blood culture	
No growth	73 (96%)
Escherichia coli	2 (3%)
Streptococcus viridians	1 (1%)
Lumbar puncture	18 (24%)
Antibiotics used	72 (95%)
Duration of antibiotics (days) (Median with IQR)	3 (2-7)
Length of NICU stay (days) (Median with IQR)	6 (3-7)

Intubation at birth was needed for 19 (25%) neonates and 12 (16%) of them required surfactant therapy. The maximum respiratory support required by the neonates was NIV (67%) followed by invasive ventilation (28%) and nasal cannula oxygen (5%). Blood culture was negative for 73 (96%) neonates after 5 days of incubation, positive for *Escherichia coli* in 2 (3%) and *Streptococcus viridians* in 1 (1%). Lumbar puncture was done in 18 (24%) neonates with all of them resulting negative after 5 days of incubation. Antibiotics were used in 72 (95%) neonates with a median duration of 3 days (IQR 2 to 7). The median duration of NICU stay was 6 days (IQR 3 to 7).

**Figure 1:**
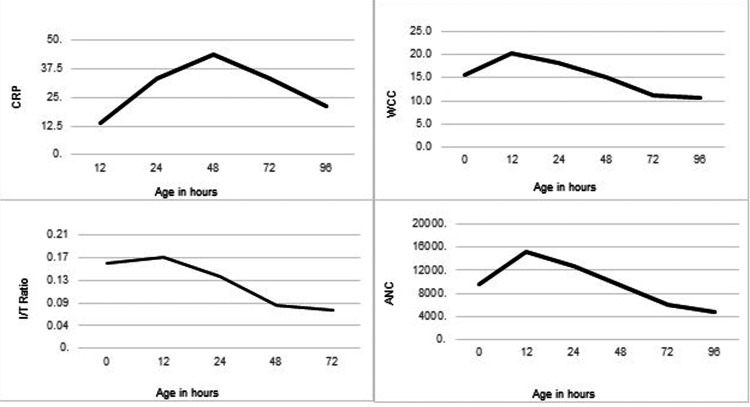
Pattern of inflammatory markers in meconium aspiration syndrome CRP, C-reactive protein; WCC, white cell count; I/T ratio, immature to total neutrophil ratio; ANC, absolute neutrophil count

Figure 1 shows the relationship between the CRP, WCC, ANC, and I/T ratio against age in hours. The CRP shows a gradual rise after birth and reaches a peak at 48 hours with an average value of 43.75 mg/L. Following the peak, the CRP falls to a value of 21.88 mg/L at 96 hours of age. The average WCC and I/T ratio showed a peak at 12 hours of age with a decline thereafter (WCC 20.4 x 103 cells/mm3 and I/T ratio 0.17). The ANC showed a similar trend with the highest average value at 12 hours of age (15349 cells/mm3).

**Figure 2:**
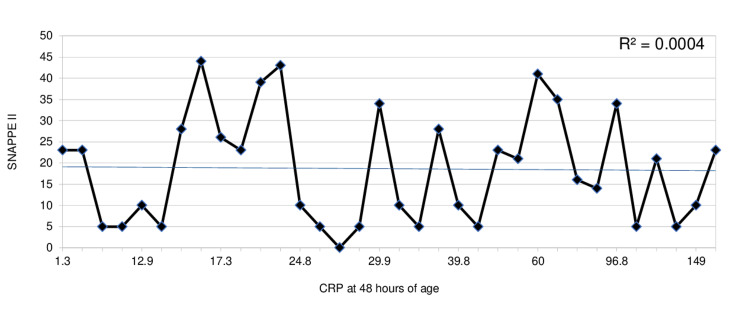
R2 for SNAPPE-II versus CRP R2, R-squared; SNAPPE-II, score for neonatal acute physiology and perinatal extension II; CRP, C-reactive protein

The correlation between CRP at 48 hours of age and SNAPPE-II score is shown in Figure 2. The R2 analysis shows that the linear correlation was low with a value of 0.0004.

Table 2 shows multiple logistic regression for CRP at different time intervals after birth against respiratory support, blood culture, use of antibiotics and SNAPPE-II score. For respiratory support, neonates who required nasal cannula had lower CRP values at 12 and 24 hours of age compared to the neonates who required invasive ventilation (-17.9, 95% CI -34.46 to -1.35, p=0.035; -33.46, 95% CI -66.33 to -0.60, p=0.046). Neonates who needed NIV as opposed to invasive ventilation had a lower CRP at 72 hours of age (-85.1, 95% CI -163.25 to -6.95, p=0.036). Neonates who had a higher CRP at 24 hours of age correlated with longer duration of antibiotics (5.80, 95% CI 2.87 to 8.73, p <0.001). There was no significant association between CRP and intubation at birth, blood culture negativity, or SNAPPE-II score.

**Table 2:** Multiple logistic regression for CRP at different ages CRP, C-reactive protein; CI, confidence interval; REF, reference variable; NC, nasal cannula oxygen; NIV, noninvasive ventilation; SNAPPE-II, score for neonatal acute physiology and perinatal extension II

Variables	CRP at 12 hours		CRP at 24 hours		CRP at 48 hours		CRP at 72 hours	
	Coefficient (95% CI)	p value	Coefficient (95% CI)	p value	Coefficient (95% CI)	p value	Coefficient (95% CI)	p value
Intubation at Birth								
Yes	-3.09 (12.31 to 6.14)	0.504	13.63 (-32.60 to 5.34)	0.1555	15.29 (-53.57 to 22.99)	0.419	29.93 (-82.25 to 22.39)	0.231
No	REF		REF	REF	REF	REF	REF	REF
Respiratory Support								
NC	-17.90 (-34.46 to -1.35)	0.035	-33.46 (-66.33 to -0.60)	0.046	-1.20 (-91.07 to 88.68)	0.978	19.60 (-28.43 to 67.64)	0.385
NIV	-9.16 (-18.41 to 0.09)	0.052	-7.57 (-26.03 to 10.89)	0.413	-3.38 (-39.66 to 32.90)	0.85	-85.10 (-163.25 to -6.95)	0.036
Invasive ventilation	REF		REF	REF	REF	REF	REF	REF
Blood Culture								
No growth	16.91 (-38.70 to 4.87)	0.125	37.80 (-81.24 to 5.63)	0.086	50.25 (-122.28 to 21.79)	0.163	39.24 (-137.62 to 59.14)	0.395
Escherichia coli	REF		REF	REF	REF	REF	REF	REF
Duration of Antibiotics	0.97 (-0.51 to 2.44)	0.195	5.80 (2.87 to 8.73)	0.000	5.97 (-0.22 to 12.16)	0.058	3.24 (-6.98 to 13.47)	0.496
SNAPPE II	-0.11 (-0.43 to 0.22)	0.509	-0.31 (-0.97 to 0.35)	0.351	-0.68 (-2.35 to 0.99)	0.409	0.54 (-1.54 to 2.63)	0.573

## Discussion

In this study of neonates with MAS, we found inflammatory markers had an early rise with a peak at 12 to 48 hours of age after birth followed by decline. CRP did not show a significant correlation with the illness severity. However, neonates who required lesser respiratory support had a lower CRP at 12 and 24 hours of age. High CRP at 24 hours led to longer duration of antibiotics, even though there was no association between CRP and the results of blood culture. There was also a trend towards longer antibiotic use when CRP was elevated at 48 hours although this did not reach statistical significance.

A large study of 162,075 term neonates found the likelihood of MAS increased with advancing gestational age at birth [22]. Our incidence of maternal GBS positivity of 32% is slightly higher than that reported by the CDC of 25% nationwide [23]. The incidence of PROM has been variable with reports from 8% to 16% [24] and maternal chorioamnionitis has been reported to be higher in neonates with MAS [25] which were similar in our group. Overall, our study population is comparable to the other cohorts with published data.

The effects of meconium following aspiration have been demonstrated in animal models [5,6]. Meconium gradually migrates from the larger to smaller airways by either spontaneous breathing or positive pressure ventilation. Meconium is cleared by macrophages in the lungs leading to inflammation which plays an important role in the pathophysiology of MAS [4].

Hofer et al. did a retrospective study excluding neonates with early onset sepsis and found that CRP and I/T ratios were higher for neonates with severe MAS during the first 2 days of life [9]. Our study showed a similar trend with WCC, ANC, and I/T ratio peaking at 12 hours and CRP peaking at 48 hours of age. However, we included the neonates with sepsis as we wanted to determine if there was a significant difference in inflammatory marker values between isolated MAS and neonatal sepsis. Although there was no linear correlation between CRP and illness severity score (SNAPPE-II), on multiple logistic regression the neonates who required nasal cannula and non-invasive ventilation had lower CRP compared to the neonates who required invasive mechanical ventilation. This is consistent with other studies that showed a significant correlation between inflammatory markers and the severity of MAS [9]. Also, this suggests a possibility that CRP could be used as a marker to predict the level of respiratory support needed for neonates with MAS.

The role of antibiotics in management of MAS is not well defined and prolonged use of antibiotics in neonates without microbial evidence of infection has been associated with adverse outcomes [11-13,15,16,26]. In our cohort, the duration of antibiotic treatment was influenced by CRP elevation at 24 to 48 hours after birth despite negative blood culture. However, our study also showed lack of correlation between CRP and blood culture result or SNAPPE-II score. Therefore, inflammatory markers alone should not be used to determine the duration of antimicrobial treatment in babies with MAS.

Our study had certain limitations. This is a single center retrospective study which lacks external validity and subject to confounding biases which could not be controlled. We excluded neonates who were transferred to other institutions and expired within 24 hours of life as we were unable to follow the course of the inflammatory markers. Including them could have changed the illness severity overall. There were only 42 neonates with CRP at 48 hours of age compared to 76 neonates at the onset of MAS. Obtaining the CRP was at physician’s discretion; hence we assume the neonates who were improving did not get further CRPs. We were not able to include procalcitonin, considered a better marker than CRP for bacterial infections [27,28] as it was not available in our institution during the study period. 

## Conclusions

We observed a pattern of inflammatory markers in MAS with a peak at 12 to 48 hours of age. Since inflammation is a natural host response to aspiration of meconium, inflammatory markers such as CRP are expected to rise in the course of illness. Thus, the utility of using CRP as an adjunctive diagnostic tool for diagnosing sepsis in babies with MAS is not recommended. This is one area of antibiotic stewardship program in the NICU that could be applied to prevent adverse outcomes associated with unjudicial and prolonged antibiotic usage in neonates.
